# Childhood Suicide Risk in the Emergency Department

**DOI:** 10.1001/jamanetworkopen.2025.22591

**Published:** 2025-07-22

**Authors:** David Pagliaccio, Jaclyn S. Kirshenbaum, Katherine M. Keyes, Randy P. Auerbach

**Affiliations:** 1 Department of Psychiatry, Columbia University, New York, New York; 2 Division of Child and Adolescent Psychiatry, New York State Psychiatric Institute, New York; 3 Department of Epidemiology, Mailman School of Public Health, Columbia University, New York, New York

## Abstract

**Importance:**

Suicide rates have increased in recent years to the third leading cause of death among youths. Children presenting to the emergency department (ED) for suicide thoughts and behaviors (STB) are at high risk for recurrent mental health (MH) concerns.

**Objective:**

To quantify rates of STB among children presenting to EDs across the US and to identify risk factors for return ED visits for STB.

**Design, Setting, and Participants:**

This cohort study examined electronic health records of children who presented to the ED from January 2010 to December 2020 using the State Emergency Department Database within the Healthcare Cost and Utilization Project. All ED visits were screened among children aged 8 to 12 years in 12 states with data linking patients across visits. *International Classification of Disease* (*ICD*) codes separated non-STB MH and STB-related visits. Data were analyzed from August 2024 to January 2025.

**Main Outcomes and Measures:**

Analyses examined characteristics of visits for MH vs STB and patients returned for subsequent ED visit(s) for STB. Analyses compared visits for MH vs. STB and examined factors related to subsequent ED return visit(s) for STB. This included sociodemographic, clinical, and temporal factors derived from electronic health records.

**Results:**

A total of 10 131 432 ED visits were screened, and 627 517 visits (6%) among 374 118 unique patients implicated *ICD* codes for non-STB MH causes (534 654 visits [5%]; mean [SD] age, 10.25 [1.41] years; 189 701 female [35%]) or STB (92 863 visits [1%]; mean [SD] age, 10.87 [1.27] years; 50 679 female [55%]). MH and STB visits increased from 36 623 ED visits (5%) in 2010 to 22 443 ED visits (10%) in 2020. Compared with non-STB MH visits, patients with STB were more likely to have private insurance and reside in areas with higher income and less deprivation (via linkage to social deprivation index). There was an age-by-sex interaction (*z* = 48.22; *P* < .001); a greater percentage of STB patients were female at older ages vs younger ages. Suicide behaviors mostly implicated injury by ingestion (7121 [43%]) or sharp or blunt object (6518 [39%]). Additionally, 68 897 patients had multiple ED visits (18%), 6537 with multiple STB visits (10%); 117 377 of 187 864 return ED visits were within 3 months (62%). STB was strongly associated with ED return for STB (aOR, 9.71 [99% CI, 9.66-9.76]; *z* = 127.93). Return STB visits within 1-year were more common for females and patients with more MH comorbidity, longer initial visits, and without private insurance.

**Conclusions and Relevance:**

Suicide risk is common but remains understudied among children. Early suicide risk was associated with recurrent STB, particularly in the months following ED discharge. Understanding risk and protective factors among children can enhance ED screening, intervention, and follow-up care.

## Key Points

**Question:**

What are the risk factors for suicide thoughts and behaviors among children aged 8 to 12 years presenting to the emergency department (ED)?

**Findings:**

In this cohort study of 627 517 electronic health records from children aged 8 to 12 years who presented to ED across 12 states from 2010 to 2020, those with suicide thoughts and behaviors exhibited sociodemographic differences from those with nonsuicide–related mental health concerns. Approximately 10% of patients with suicide risk returned for multiple suicide-related ED visits.

**Meaning:**

These findings suggest that suicide risk is common and recurrent among children in the ED, underscoring the importance of screening, intervention, and follow-up care for these high-risk youth.

## Teaser

This cohort study examines electronic health records from children presenting to the emergency department (ED) for suicide thoughts and behaviors to identify risks of return ED visits.

## Introduction

Despite varied screening and prevention efforts, suicide is a leading cause of death among youth, with rates increasing^1^ particularly among racially and ethnically minoritized populations.^2,3,4^ Increasingly, suicide thoughts and behaviors (STB) have become a public health concern among preadolescent children (eg, children aged 8 to 12 years),^5,6,7^ and more screening and prevention may be warranted to support this age group. The emergency department (ED) often serves as the primary point-of-contact for youth to receive psychiatric care for STB. Among adolescents (ie, aged 13 to 18 years) receiving inpatient or ED care for STB and/or other psychiatric concerns, approximately 10% will attempt suicide and approximately 30% will exhibit broader suicide behaviors within 3 to 6 months of discharge.^8,9,10,11^ However, the severity and time-course of clinical outcomes for younger children remain unclear.

In contrast to a robust literature on adolescent suicide risk (ie, aged 13 to 18 years), work in preadolescent youth (ie, aged 8 to 12 years) is limited. To address this critical gap, this cohort study examined STB in children aged 8 to 12 years by harnessing the strength of the Healthcare Cost and Utilization Project (HCUP), a large database of electronic health records (EHR) from across the US supported by the Agency for Healthcare Research and Quality.^12,13,14^ Specifically, we examined the State Emergency Department Databases (SEDD), which catalog millions of person-level hospital-affiliated ED visits.^12^ EHR have been underused to study suicide in children, despite promising work in adults and adolescents. Our goal was to characterize the rate and timeline of return ED visits for STB among children presenting to the ED and to examine sociodemographic and clinical factors associated with ED visits for STB and that were associated with ED return. First, we hypothesized that, compared with nonsuicide–related mental health (MH) cases, children with STB would be more likely to be female, have private insurance,^15^ report greater internalizing comorbidities^6^; and that ingestions would be the main injury method.^16,17^ Second, consistent with prior research, we anticipated seasonal trends in STB (eg, more cases during the school year).^15^ Last, we hypothesized that children with STB at an initial visit, female children, and those with more psychiatric comorbidities would be most likely to return to the ED.^18^ Analyses focused on factors that differentiate STB from non-STB MH cases (rather than physical health) to advance our understanding of psychiatric ED care to mitigate suicide risk in children.

## Methods

### Data Overview

HCUP SEDD^12^ Core data were analyzed from August 2024 to January 2025 via Redivis,^19^ as approved by the Columbia University institutional review board. Informed consent was not required because data were deidentified. Variables linking ED visits for individual patients over time^20^ were available for 12 states: Arkansas, 2013 to 2020; Florida, 2010 to 2014 and 2016 to 2020; Iowa, 2010 to 2019; Indiana, 2017 to 2019; Maryland, 2013 to 2020; Massachusetts, 2010 to 2019; Missouri, 2017 to 2019; Nebraska, 2010 to 2019; New York, 2010 to 2018; Utah, 2010 to 2019; Vermont, 2011 to 2019; and Wisconsin, 2013 to 2020. Visits were filtered to retain only children aged 8 to 12 years (inclusive) at the time of ED admission; visits without *International Classification of Diseases* (*ICD*) diagnostic codes were excluded. The data workflow is summarized in eFigure 1 in Supplement 1. Methods and results follow the Reporting of Studies Conducted Using Observational Routinely-Collected Data (RECORD)^21^ extension of Strengthening the Reporting of Observational Studies in Epidemiology (STROBE) reporting guideline (eMethods in Supplement 1).

### Variable Creation

Visits were labeled for main analyses based on *ICD* Ninth Revision (*ICD-9*) and *ICD* Tenth Revision (*ICD-10*) diagnostic code(s) and/or *ICD-9* E codes into 2 groups: nonsuicide–MH visits vs visits with STB. Code selection built on prior work and HCUP guidance (eMethods in Supplement 1).^22^ The HCUP separates 2015 into 2 datasets—quarters 1, 2, and 3 of 2015 included *ICD-9* codes and quarter 4 which began the implementation of *ICD-10* on October 1, 2015. Visits for physical health reasons only were excluded; the total number of visits for any reason were tabulated per state per year for contextualization. Codes with unclear intent were explored as potential suicide behaviors but not used in main analyses because the nature of these events could not be confirmed definitively from HCUP records alone. Supplementary analyses split visits based on indication of suicide ideation vs suicide behavior for additional specificity.

### Covariates and Estimators

Estimators and covariates were selected from common data elements available across states and years: age at admission, sex, insurance status (private or not), disposition (routine discharged home or not). HCUP provides mutually exclusive indication of race or ethnicity logged in EHR; for analyses, we examined Black, Hispanic, and White identities, relative to other identities (Asian or Pacific Islander, American Indian and Alaska Native, other, missing). Given small cell sizes for these identities, we collapsed these races and ethnicities into the other category. The urbanicity of patient location was coded by HCUP based on Core-Based Statistical Area and binarized for analysis as metropolitan area (more than 1 million residents) or not. Local socioeconomic status was examined based on median household income by patient zip code (binarized as lowest quartile in the state vs higher 3 quartiles). The length of ED stay was examined as a continuous outcome and binarized as 1 day vs longer visits. The total monetary charges for the visit were examined as a continuous outcome and binarized as more vs less than $2500 (approximately the 80th percentile). The presence of comorbid internalizing (depressive or anxiety disorder) or externalizing (attention-deficit/hyperactivity [ADHD], oppositional defiant disorder, conduct disorder) disorders were ascertained from the top 5 *ICD* codes. The first 3 *Current Procedural Terminology* (*CPT*) codes were examined to identify high complexity visits requiring comprehensive history or examination, high medical decision-making, and severe presenting problems (99285).

In additional analyses, patient zip codes were linked to the 2019 Social Deprivation Index (SDI).^23,24^ Not all hospitals, states, or years provided zip code data to HCUP for analysis (missing data were dropped listwise from these analyses); sample sizes are noted for analyses with missing data. This SDI is a 1 to 100 composite score of 7 social determinants of health from the American Community Survey; (eg, poverty, education, housing, and unemployment). Supplementary analyses explored the 2018 Area Deprivation Index^25^ and 2015 Child Opportunity Index 2.0.^26,27^ Timing of ED visits was examined based on admission on weekend vs weekday, in summer (June, July, August vs not), and during the day and evening (9 am to 9 pm vs not).

Other *ICD* codes were explored but lacked sufficient sample size for well-powered analyses: nonsuicidal self-injury (NSSI),^28^ sleep disorders or disturbance,^29^ sexual and gender identity disorders,^30,31^ housing or family circumstances,^32^ and substance use disorders^33^ (eMethods in Supplement 1). Similarly, HCUP coding of point of origin for admission or visit was explored but was limited by sample size for analysis.

### Statistical Analyses

Given the large sample size, analyses focused on effect size and 99% CIs (all significance tests are 2-sided). Statistical analyses were conducted in R version 4.4.1 (R Project for Statistical Computing) with tidyverse.^34,35^ Patient characteristics were compared across groups (non-STB MH vs STB) using the scipub package.^36^ Continuous characteristics were compared across groups with *t* tests and Cohen *d* effect size. Binary characteristics were compared with χ^2^ tests and converted to an odds ratio (OR). MH and STB visits were totaled per state per year and rates were estimated per state population of children aged 5 to 14 years in the 2020 US Census to compare per capita rates across states (eMethods in Supplement 1). Trends over time were examined using Kendall τ rank-correlation.

Logistic regressions were used to examine multiple factors associated with group and likelihood of ED return. Regression results included SE estimates. Adjusted odds ratios (aOR) were presented for logistic regressions, controlling for covariates; aOR were unstandardized for continuous variables (eg, age in years). Sensitivity models probed the robustness of these models to potential state differences with mixed-effects model (random intercept per state) and leave-1-state-out cross-validation approaches.

## Results

### Overview

A total of 10 131 432 ED visits were available for children aged 8 to 12 years across states and years. Of these, 627 517 (6%) visits for coded for MH and/or STB. This included 534 654 MH visits (85%), with a mean (SD) age of 10.25 (1.41) years and 189 701 females (35%), and 92 863 STB visits (15%), with a mean (SD) age of 10.87 (1.27) years and 50 679 females (55%). Common reasons for MH visits included conduct disorder, ADHD, anxiety, and emotional symptoms. Across states, the per capita rate of STB visits (per Census population of children ages 5 to 14 years) tended to increase year-over-year (Kendall τ = 0.53, *df* = 97; *z* = 7.40; *P* < .001) (Figure, A and eTable 1 and eFigure 2 in Supplement 1). STB visits showed particular increases over time (proportion of STB out of all MH and STB visits: Kendall τ = 0.59; *df* = 97; *z* = 8.26; *P* < .001) (Figure, B). This corresponded to an increase in STB-related patient charges over time (Kendall τ = 0.35; *df* = 97; *z* = 4.87; *P* < .001) (Figure, C), totaling more than $50 million in 2018 alone. Rates of *ICD *code groups by state are displayed in eFigure 3 in Supplement 1.

**Figure.**
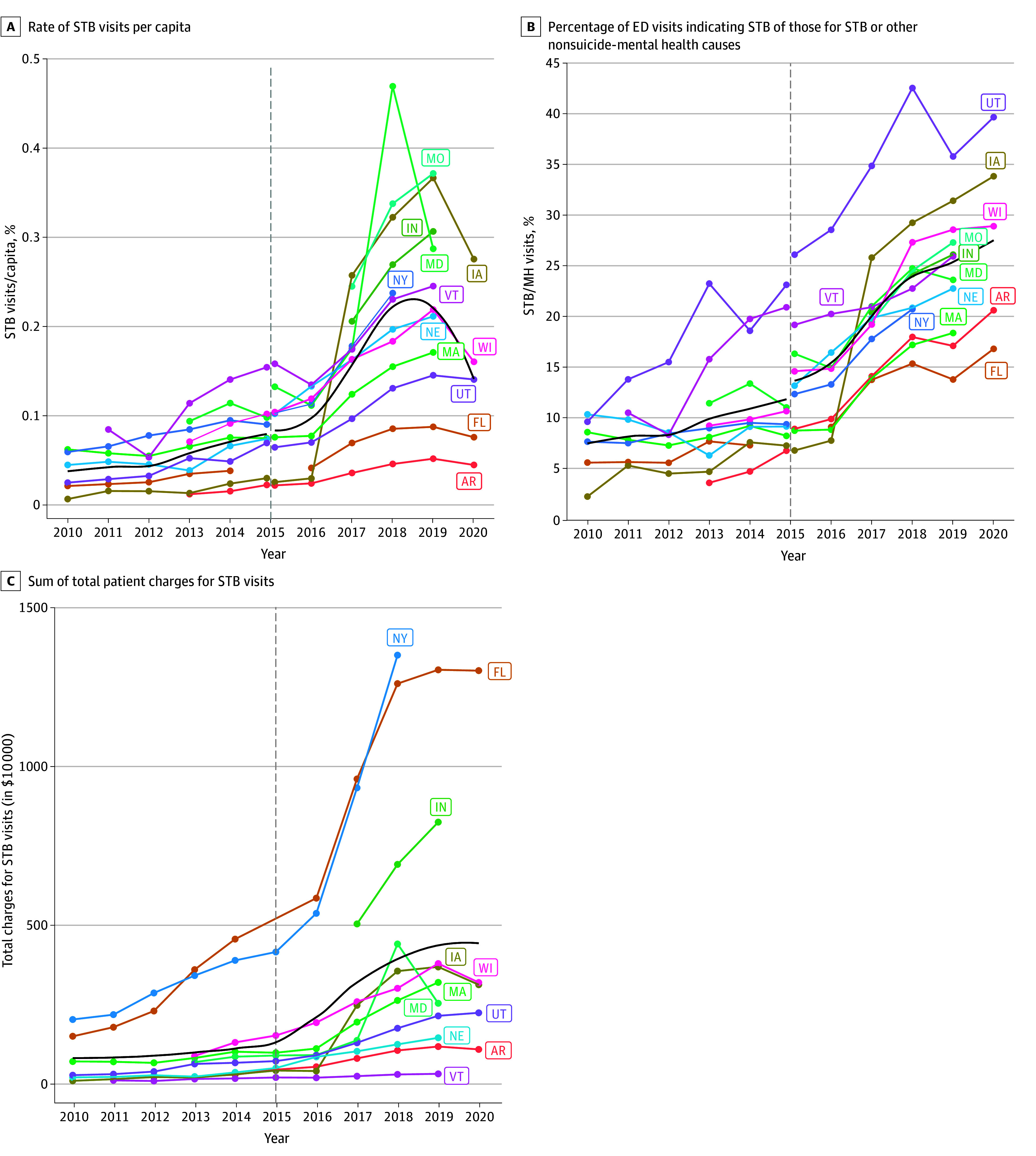
Change in Suicide Thoughts or Behaviors (STB)–Related Emergency Department (ED) Visits by State Over Time The number of visits for STB were tabulated per state (of ED) and per year (of admission date). Panel A displays the rate of STB visits per capita, adjusted by the state population of children (5 to 14 years old) in the 2020 US Census. Panel B displays the percentage of ED visits indicating STB out of those for STB or other nonsuicide–mental health causes. Panel C displays the sum of total patient charges for STB visits (when available in the dataset) scaled by $10 000. The dashed line at 2015 indicates change from *International Classification of Diseases, Ninth Revision *(*ICD-9*) to *Tenth Revision* (*ICD-10*) (2 points are indicated per state at 2015 to separate *ICD-9* in quarters 1 to 3 and *ICD-10* in quarter 4 in Panels A-B). The black line shows a loess fit line across states. AR indicates Arkansas; FL, Florida; IA, Iowa; IN, Indiana; MA, Massachussetts; MD, Maryland; MO, Missouri; NE, Nebraska; NY, New York; UT, Utah; VT, Vermont; WI, Wisconsin.

### Sociodemographic Group Differences

MH and STB visits showed significant differences across sociodemographic factors (Table 1). Compared with MH visits, patients presenting with STB tended to be slightly older; were more likely to be female, be White, have private insurance, and live in higher income areas; and were less likely to live in metropolitan areas. Similar patterns were observed when comparing visits coded for suicide behavior to those with suicide ideation (eTable 2 in Supplement 1). In a logistic regression examining group status (STB vs MH), there was an age-by-sex interaction (OR, 1.32 [99% CI, 1.31-1.35]; SE = 0.005; *z* = 48.22; *P* < .001; n = 627 506) (eFigure 4 in Supplement 1), such that sex differences in STB were more pronounced at older compared with younger ages. Specifically, there was no sex difference at age 8 years (MH visits: 23 930 of 82 751 children were females [29%]; STB visits: 1936 of 6703 females [29%]; OR, 1.00 [99% CI, 0.93-1.07]), whereas STB patients were disproportionately female at age 12 (MH visits: 59 745 of 137 704 [43%]; STB visits: 27 388 of 40 461 [68%]; OR, 2.73 [99% CI, 2.65-2.82]). Overall, there was a similar count of STB visits for male and female patients at younger ages but double the number of female vs male STB patients at age 12 years (eFigure 4 in Supplement 1). Demographic trends over time for STB visits are shown in eFigure 5 in Supplement 1.

**Table 1.** Characteristics of MH and Suicide-Related Visits^a^

Variable	Visits, No. (%)		χ^2^	OR (99% CI)
	Non-STB MH (n = 534 654)	STB (n = 92 863)		
Sociodemographics				
Age, mean (SD), y	10.25 (1.41)	10.87 (1.27)	134.11^b^	*d* = 0.44 (99%CI, 0.44 to .0.45)^b^
Sex				
Female	189 701 (35)	50 679 (55)	12 207.01	2.18 (2.14 to 2.23)
Male	344 948 (64)	42 178 (45)	1 [Reference]	1 [Reference]
Race				
Black	112 933 (22)	16 454 (19)	515.71	0.81 (0.79 to 0.83)
White	283 679 (55)	52 389 (59)	479.02	1.18 (1.15 to 1.20)
Ethnicity				
Hispanic	75 241 (15)	11 561 (13)	153.84	0.88 (0.78 to 0.82)
Non-Hispanic	439 577 (85)	77 150 (87)	1 [Reference]	1 [Reference]
Insurance				
Private	155 333 (29)	30 543 (33)	560.58	1.20 (1.17 to 1.22)
Not private	378 761 (71)	62 206 (67)	1 [Reference]	1 [Reference]
Median household income in zip code (lowest quartile)				
Lowest quartile	194 817 (37)	29 254 (32)	854.14	0.80 (0.79 to 0.83)
3 Upper quartiles	335 042 (63)	62 860 (68)	1 [Reference]	1 [Reference]
Neighborhood Deprivation [SDI; 1-100], mean (SD)	57.9 (29.42)	53.09 (29)	−42.77^b^	*d* = −0.16 (99% CI, −0.16 to −0.17)^b^
Urbanicity				
>1 Million residents	281 727 (54)	46 169 (50)	285.22	0.89 (0.87 to 0.90)
≤1 Million residents	251 918 (47)	46 556 (50)	1 [Reference]	1 [Reference]
Clinical factors				
No. of diagnoses, mean (SD)	2.82 (1.6)	2.94 (1.85)	19.79^b^	*d* = 0.08 (99% CI, 0.07 to 0.08)^b^
Diagnosis				
Internalizing	130 728 (24)	37 900 (41)	10 777.93	2.13 (2.09 to 2.17)
Externalizing	220 029 (41)	19 883 (21)	13 057.88	0.39 (0.38 to 0.40)
Length of ED stay, mean (SD), d	0.19 (0.54)	0.40 (0.73)	85.49^b^	*d* = 0.37 (99% CI, 0.37 to 0.38)^b^
Length of ED stay (>1 d)	83 807 (16)	30 512 (33)	15 693.81	2.63 (2.58 to 2.69)
Disposition (discharge home)	439 579 (92)	51 597 (60)	65 874.02	0.13 (0.13 to 0.13)
Total charges, mean (SD), $	1772.16 (2774.5)	2515.35 (2651.97)	75.20^b^	*d* = 0.27 (99% CI, 0.26 to 0.28)^b^
Total charge (>$2500)	84 471 (17)	28 661 (34)	13 702.58	2.54 (2.49 to 2.60)
High complexity visit	54 972 (10)	34 271 (37)	45 968.52	5.10 (5.00 to 1.76)
Temporality of admission				
On weekday (Monday to Friday)	413 102 (77)	79 288 (85)	3082.48	1.72 (1.68 to 1.76)
Daytime hours 9 am to 9 pm	279 788 (72)	43 520 (77)	550.18	1.28 (1.25 to 1.32)
School months (September to May)	300 761 (78)	59 042 (85)	1402.36	1.52 (1.47 to 1.56)
School day (weekday September to May)	236 927 (56)	50 959 (70)	4516.06	1.77 (1.74 to 1.81)

Abbreviations: MH, mental health; OR, odds ratio; SDI, social deprivation index; STB, suicide thoughts or behaviors.

^a^ Sociodemographic, clinical, and temporal characteristics of pediatric visits are presented. All differences between groups were *P* < .001 significant comparing visits with STB with MH visits without STB. Categorical variables are summarized by count and subgroup percentage. Group differences were tested via χ^2^ test. OR are presented for effect size such that positive OR indicate more likelihood of being in the comparing visits with STB to MH visits without STB group for the indicated category (eg, female > male for sex). Effect sizes are presented with 99% CI. Missing data per variable: sex (n = 11), race or ethnicity (n = 23 988), insurance (n = 674), income (n = 5544), urbanicity (n = 1147), admission date (n = 74), length of stay (n = 981), disposition (n = 65 366), total charges (n = 33 885), admission day (n = 74), admission time (n = 182 053), admission month (n = 173 240), and SDI (n = 121 438; zip codes were unavailable for Arkansas and Massachusetts in all years and Maryland in 2013 to 2017).

^b^ Continuous variables are summarized with their group mean (SD). Group differences were tested via *t* test with their accompanying Cohen *d* effect size. Positive Cohen *d* indicate higher continuous score in the STB relative to the MH group.

### Neighborhood Deprivation

STB visits were associated with lower neighborhood deprivation (SDI in their home zip code) than MH visits (Table 1). This difference persisted in a logistic regression when covarying for personal demographics (age, sex, race or ethnicity, and insurance) and comorbidity (*b* = −2.17 [99% CI, −2.37 to −1.96]; SE = 0.105; *z* = −20.59; Cohen *d* = −0.087; N = 482 010). In a logistic regression, a 10-point (1 decile) higher SDI was associated with a lower likelihood of an STB visit vs MH visit (aOR, 0.97 [99% CI, 0.96 to 0.97]; SE = 0.002; *z* = −20.34; *P* < .001; n = 482 010).

### Clinical Group Differences

Compared with MH visits, patients with STB were assigned more *ICD* diagnostic codes, which may in part be associated with the use of injury codes (Table 1). Patients with STB were more likely to exhibit internalizing disorders whereas externalizing disorders accounted for more MH cases. Comorbid internalizing and externalizing diagnoses were also more common among STB than MH cases (OR, 1.34 [99% CI, 1.29 to 1.38]; *P* < 001). STB cases were more likely to have ED stays of more than 1 day, high complexity visit CPT codes, more costly charges, and were less likely to be discharged home than MH cases. Similar patterns were observed when comparing visits coded for suicide behavior with suicide ideation (eTable 2 in Supplement 1).

### Exploratory Variables

Of 346 988 *ICD-10* MH and STB cases, family or housing circumstances were coded in only 5808 (1.7%) of cases and NSSI or history of self-harm was coded in 4954 (1.4%) of cases. Both factors were more commonly used among STB than MH cases (family OR, 2.64 [99% CI, 2.45 to 2.83]; NSSI OR, 6.87 [99% CI, 6.37 to 7.42]).

### Timing of Admissions

Compared with MH visits, STB admissions were more likely to occur during daytime hours (9 am to 9 pm), during the school year (September to May), and on weekdays. Overall, STB visits were more common during typical school days (nonsummer weekdays) (Table 1 and eTable 2 and eFigure 6 in Supplement 1). Note that 328 365 of 350 786 visits (94%) with a logged point of origin indicated a nonhealth care facility point of origin (ie, including routine self-referral or physician referral). Specifics about potential referrals from school were not available.

### Ambiguous *ICD* Codes

Injury with undetermined intent and sequalae of self-harm codes that potentially reflect suicide behaviors were present for 2529 MH cases (1%) and 2265 cases with suicide ideation (3%) (not examining cases with explicit codes for suicide behavior). Among suicide ideation visits, these ambiguous codes were more likely to be used for patients who were older, female, and with more internalizing disorders (eTable 3 in Supplement 1). These visits also tended to be longer, more costly, and more likely to be high complexity visits. Patients with these codes at their first visit with suicide ideation were more likely to have another visit for STB than patients at other suicide ideation visits 192 of 1626 visits with ambiguous codes (12%) vs 5243 of 55 858 visits without (9%) (OR, 1.29 [99% CI, 1.05-1.58]; χ^2^ = 10.54; *P* = .001).

### Injury Methods

Of the 16 751 suicide behavior visits, 7121 (43%) included codes for injury by ingestion and 6518 (39%) for injury by sharp or blunt object. Other methods were rarely specified, (eg, firearms (34 [0.2%]), jumping (107 [0.6%]), motor vehicles (88 [.5%]), or asphyxiation, such as drowning, hanging, or suffocation (540 [3%]); 191 included multiple injury codes (1%). Compared with object-based injury visits, ingestion was more common among patients who were older, female, on private insurance, and in higher income and less urban areas (eTable 4 in Supplement 1). Ingestion cases also exhibited less comorbidity, but were longer, more complex, and more costly ED visits. Sample sizes were limited for in-depth analysis of other methods, but these methods were disproportionately present among male (477 of 2978 males [16%]) relative to female suicide behavior cases (277 of 11049 females [3%]), and nearly all firearm injuries were among males.

### Repeat ED Visits

Among 627 517 MH and STB visits, there were 374 118 individual patients. Most patients (305 221 [82%]) had only 1 ED visit in the dataset; 68 897 (18%) had more than 1 visit (39 724 [11%] with 2 visits; 13 934 [4%] with 3 visits, 15 239 [4%] with 4 or more visits). Among return visits, 89 002 (47%) occurred within 1 month of the prior ED visit, 117 377 (62%) within 3 months, and 162 082 (86%) within 1 year (eFigure 7 in Supplement 1). Among patients with STB, 6537 (10%) had multiple STB visits.

In logistic regressions (Table 2), STB at a given visit was the strongest estimator of a return ED visit for STB within 1 year (aOR, 9.71; 95% CI, 9.66-9.76) 64% of STB returns followed a prior STB vs MH visit). Returns within 1 year for STB were more common for patients who were female, non-Hispanic, did not have private insurance, lived outside metropolitan areas, had more MH comorbidity, had longer initial visits, and had visits that did not discharge home. Results were robust in sensitivity analyses (eTable 5 in Supplement 1). Among 11 586 visits for suicide behaviors, object-based injury was associated with higher odds of return for STB within 1 year (aOR, 1.28 [99% CI, 1.14-1.42]; SE = 0.055; *z* = 4.40; *P* < .001) relative to ingestion injury visits, adjusted for all covariates listed previously.

**Table 2.** Factors Associated With Return to ED for STB Within 1 Year^a^

Variable	Model 1: all visits		Model 2: STB Visits	
	aOR (99% CI)	*z*	aOR (99% CI)	*z*
STB vs MH	9.71 (9.66-9.76)	127.93	NA	NA
Age, y	1.06 (1.04-1.08)	9.25	0.99 (0.97 to 1.01)	−1.30^b^
Female vs male	1.33 (1.29-1.37)	17.95	1.26 (1.21-1.31)	11.00
Race				
White	1.05 (0.98-1.12)	1.89^b^	1.19 (1.10-1.28)	4.86
Black	1.03 (0.95-1.11)	0.98^b^	1.08 (0.97-1.19)	1.94^b^
Hispanic vs non-Hispanic	0.72 (0.63-0.81)	−9.12	0.78 (0.66-0.90)	−5.28
Private insurance	0.79 (0.74-0.84)	−13.28	0.78 (0.72-0.84)	−11.32
Urbanicity, >1 million residents	0.68 (0.64-0.72)	−23.76	0.57 (0.51-0.63)	−26.58
Diagnosis				
Internalizing	1.37 (1.33-1.41)	19.66	1.17 (1.12-1.22)	8.01
Externalizing	1.39 (1.35-1.43)	19.35	1.42 (1.36-1.48)	15.18
Length of ED stay (>1 d)	1.20 (1.15-1.25)	10.31	1.15 (1.10-1.20)	6.47
Disposition (discharge home)	0.71 (0.66-0.76)	−18.09	0.83 (0.78-0.88)	−8.64
High complexity visit	1.13 (1.08-1.18)	6.37	1.00 (0.95-1.05)	0.20^b^

Abbreviations: aOR, adjusted odds ratio; ED, emergency department; NA, not applicable; STB, suicide thoughts or behaviors.

^a^ Logistic regression models tested whether each visit was followed by a subsequent visit for STB within the next 1 year. Cases were removed with listwise deletion for missing covariates. Model 1 examined 537 043 visits, with 3.73% having a return visit for STB within 1 year. Model 2 examined only STB visits (n = 80 956), with 16.13% having a 1-year return for STB. All factors were *P* < .001 significant unless denoted as not significant. The aOR, 99% CI, and *z* statistic are presented for each variable.

^b^ Not significant.

## Discussion

This cohort study examined large-scale EHR data from children aged 8 to 12 years across 12 states in the HCUP SEDD (2010 to 2020). Among 10 million records, more than 600 000 ED visits (6%) indicated MH and/or suicide-related concerns. ED visits with STB increased over time for all states examined, particularly from 2016 to 2020, both per capita and relative to non-STB MH visits. This corresponded to large increases in total patient charges over time. Despite rising STB rates, work suggests that suicide deaths (from official records) are relatively uncommon among children aged 5 to 14 years, with many more nonfatal attempts requiring ED or other hospitalizations.^17^

### Sociodemographic Differences

Compared with other MH visits, children with STB were more likely to be older, female, White, on private insurance, and live in higher income but less metropolitan areas. These sex and race or ethnicity differences between groups may also be increasing over time, particularly in the last several years. Further, sex differences emerged across age—STB cases were disproportionately female at older ages. This likely represents an increase in STB risk for girls throughout development (eg, with onset of puberty).^37,38,39,40^ Non-STB MH visits remained predominantly male, which may also be associated with developmental increases in other issues (eg, conduct disorder or attention problems).^41,42^ Prior work also suggests that youth with STB are more likely to be female whereas sex differences were less apparent among adults presenting to the ED with STB.^15^ This less pronounced sex difference among adults may be associated with more severe attempts or fatal suicides among adult males.^43^ Studies have further suggested increases in suicide risk among Black and Hispanic youth.^1,4^ In the current data, STB cases were slightly more likely to involve White patients and non-STB MH cases were more likely to be Black or Hispanic. This may be limited by the states available for analysis and may underrepresent the full national picture. Disparities may also be more evident among more severe cases or among suicide deaths.^43,44^

Findings corroborate prior risk associated with family or housing disruptions,^32^ although use of these *ICD* codes was rare and may be better captured through more detailed assessment methods. STB cases were more common among suburban populations (ie, less in high-population urban centers but more in higher income areas), compared with non-STB MH cases. STB visits exhibited lower neighborhood deprivation (SDI). Results were matched with alternative metrics of area deprivation.^45,46^ Alternatively, other work has suggested that children from more disadvantaged neighborhoods have higher ED use and more hospitalization, particularly for conditions that may be more cost-effectively managed in primary care or other settings.^47,48^ Importantly, note that HCUP data did not include individual measures of household income or urbanicity. Nonetheless, expanding access to non-ED care (eg, community health centers) can help reduce psychiatric ED visits for youth and may close racial and socioeconomic disparities in ED use.^49,50^

### Timing of Admissions

When examining the temporality of ED admissions, we identified several key patterns, building on prior work. Most MH and STB visits occurred during the week (Monday through Friday) compared with weekends, but this was disproportionately so among STB visits. Similarly, most visits occur during school months (September through May),^15,51,52^ particularly in the spring (March through May) and less so in the summer (June through August). These trends were magnified among STB visits with additional increases in the fall (October and November). Finally, compared with MH visits, STB admissions were more likely to occur during the day and evening (9 am to 9 pm) than late night or early morning. Patterns may differ from those in adults (eg, peaks of suicide attempts in the spring),^53,54^ or inpatient admissions for suicide and self-inflicted injury being highest in summer and on weekends.^55^ HCUP data did not include nuanced coding of potential school-based referral to the ED, which could be examined in future work to understand these differences in timing of admission.

### Injury Methods

Suicide behavior predominantly included ingestion (ie, overdose) or object-based injuries (eg, cutting).^17^ Ingestions tended to incur longer, more complex, and more costly ED visits. Object-based injuries may be associated with greater likelihood of return ED visits for STB. Firearm and other injury causes were rare but disproportionately present among males.^44^ Rates of self-harm and suicide by firearm are substantially higher among adults than children,^56^ and firearms are the most lethal suicide method.^57^ Reducing access to lethal means is a critical intervention point; psychoeducation on home sanitization via hospital or ED clinical staff can help mitigate future suicide risk.^58^ Given the preponderance of injuries by ingestion or objects, parent education programs^59^ about safe storage (eg, lock boxes) for medications, cleaning supplies, and sharps can reduce children’s access to lethal means.

### Repeat ED Visits

Many children (18%) used ED services repeatedly for MH and STB care; 10% of children with STB had multiple STB-related visits. Most returns to the ED occurred within the first several months after discharge. STB were the strongest estimator of a return ED visit for STB within 1 year, similar to prior work.^18^ Risk for return may also be higher among patients who were female, non-Hispanic, without private insurance,^60^ outside metropolitan areas, and more clinically severe. Estimative models using adult EHR often highlight other concerns, such as substance use, or physical health problems not examined herein.^60,61^ Lack of regular care from an MH care professional (or change in care) is shown to increase likelihood of seeking emergency services.^62,63^ Understanding the availability of MH clinicians in a given area could aid in future analyses of ED return rates. Work suggests that adults on public insurance may be more likely to seek ED care.^63,64,65^ To avoid overtaxing ED resources for nonemergency causes, more resources and policy supports are needed to reduce barriers to youth MH care throughout a variety of settings (eg, primary care, outpatient clinics, and school psychologists).^66,67,68,69^

### Limitations

This study has limitations. Data were limited to structured variables in HCUP EHR. Nuances of suicide risk can be assessed in clinical notes that may be available in other EHR data^60^ or through prospective studies.^70^ Data covered the 2015 transition from *ICD-9 *to* ICD-10*. Differences in the number and specificity of suicide-related codes in *ICD-9* and *ICD-10* may partially account for some changes in prevalence but are unlikely to fully account for the observed changes. Similarly, there may be state-specific or hospital-specific differences in coding practices that are difficult to ascertain from EHR. Determining the validity of self-injury and suicide codes can be challenging^71,72^ and may be limited by stigma around suicide.^73^ Work suggests that coding may be more accurate with precise *ICD-10 *codes^74^ but also may depend on context or methods.^75,76^ These factors likely contribute to an underreporting or undercoding of STB for youth.^77,78^ Thus, we anticipate that observed STB rates and STB vs non-STB MH differences may be an underestimation (ie, cases with STB codes were likely to be true positives but false negatives were likely in the MH group). Similarly, we highlighted a small but important selection of cases with ambiguous codes (ie, injury with undetermined intent). Without further corroboration from more in-depth clinical assessment, these cases cannot be definitely identified as suicide-related or not, although they do exhibit similar characteristics to STB cases. Codes were limited for NSSI and other factors of interest, limiting statistical power. A specific *ICD-10* code for NSSI was implemented in 2021, which may facilitate future EHR analyses. Analysis of neighborhood deprivation was limited by whether states contributed patient zip codes and lack of access to patient addresses or census tracts that may better characterize local environment (or individual family-level socioeconomic status).^45,46^

## Conclusions

Despite these increasing rates, suicide remains an understudied concern among children.^7^ Many preadolescent children present to the ED with suicide risk, with rates increasing in recent years. These children exhibit greater psychiatric comorbidity and incur greater financial costs in the ED compared with children presenting for other MH causes. The ED is a critical point-of-contact for initiating psychiatric evaluation and care for many youth. Yet, the months after ED discharge are a high-risk period for reemergence of STB. EHR have been an underused data source that can help inform large-scale patterns in STB and ED care. Future studies combining structured EHR data, narrative clinician notes, and prospective assessment can enhance understanding of suicide risk and recurrence among children in the ED. This is an important step toward tackling the public health crisis of youth suicide and to improve screening and actionable preventative intervention approaches.

## Notes

Supplement 1.
eMethods.
**eFigure 1. **Workflow **eFigure 2. **Count of Suicide-Related Visits Per State Per Year **eFigure 3.** Rates of Code Groups by State **eFigure 4.** Age × Sex Differences **eFigure 5.** Demographic Trends in Suicide-Related Visits over Time **eFigure 6.** Visit Timing **eFigure 7.** Timing of ED Return Visits **eTable 1.** Count of Visits Per State Per Year **eTable 2.** Characteristics of Suicide-Related Visits **eTable 3.** Characteristics of SI Visits with Ambiguous Behavior Codes **eTable 4. **Characteristics of Suicide Behavior Visits by Method **eTable 5.** Sensitivity Analyses for State Differences

Supplement 2.
Data Sharing Statement
