# Supplementary material for: Childhood Suicide Risk in the Emergency Department
Source: JAMA Netw Open. 2025 Jul 22;8(7):e2522591. doi: 10.1001/jamanetworkopen.2025.22591 (PMC12284741; doi:10.1001/jamanetworkopen.2025.22591)
Supplement: Supplement 1. — eMethods. eFigure 1. Workflow eFigure 2. Count of Suicide-Related Visits Per State Per Year eFigure 3. Rates of Code Groups by State eFigure 4. Age × Sex Differences eFigure 5. Demographic Trends in Suicide-Related Visits over Time eFigure 6. Visit Timing eFigure 7. Timing of ED Return Visits eTable 1. Count of Visits Per State Per Year eTable 2. Characteristics of Suicide-Related Visits eTable 3. Characteristics of SI Visits with Ambiguous Behavior Codes eTable 4. Characteristics of Suicide Behavior Visits by Method eTable 5. Sensitivity Analyses for State Differences [file jamanetwopen-e2522591-s001.pdf]

Supplemental Online Content

Pagliaccio D, Kirshenbaum JS, Keyes KM, Auerbach RP. Childhood suicide risk in the emergency department via the Healthcare Cost and Utilization Project. *JAMA Netw Open.* 2025;8(7):e2522591. doi:10.1001/jamanetworkopen.2025.22591

eMethods.
eFigure 1. Workflow
eFigure 2. Count of Suicide-Related Visits Per State Per Year
eFigure 3. Rates of Code Groups by State
eFigure 4: Age × Sex Differences
eFigure 5. Demographic Trends in Suicide-Related Visits over Time
eFigure 6. Visit Timing
eFigure 7. Timing of ED Return Visits
eTable 1. Count of Visits Per State Per Year
eTable 2. Characteristics of Suicide-Related Visits
eTable 3. Characteristics of SI Visits with Ambiguous Behavior Codes
eTable 4. Characteristics of Suicide Behavior Visits by Method
eTable 5. Sensitivity Analyses for State Differences

This supplemental material has been provided by the authors to give readers additional information about their work.

© 2025 Pagliaccio D, et al. *JAMA Network Open*.

eMethods.

Overview: HCUP provides deidentified variables to analyze ED visits and returns: a verified patient linkage ID (visitLink) and the relative timing of ED intake (DaysToEvent). This is available for 17 states; data from 12 states were available to Columbia University researchers. Repeat visits within this dataset were examined and the subset of repeat visits for STB were also quantified. The days between repeat visits were quantified. The main reason(s) for visit and top 5 ICD codes were examined for MH concerns. The main reason(s) for visit and top 15 ICD codes were screened for STB (for a more inclusive identification of suicide risk). Suicide ideation included one code per version (ICD-9: V62.84; ICD-10: R45.981). Suicide behaviors include ICD-9 injury e-codes (E95.1-E95.8), one ICD-10 code for suicide attempt (T14.91), and ICD-10 codes for initial encounters for intentional self-harm derived from prior HCUP definition.

STB Rates over Time: MH/STB were totaled per state-year and normalized by state population of children (5-14-years-old) in the 2020 U.S. Census (https://data.census.gov/) to compare per capita rates across states:

AR=929175, FL=2414374, IA=423503, IN=907525, MA=772889, MD=778153,
MO=786205, NE=276231, NY=2319613, UT=544452, VT=68018, WI=733363

For data in 2015, state counts using ICD-9 in the first 3 quarters of the year were normalized by ¾ of the state population and ICD-10 data in 2015 Q4 were normalized by ¼ of the state population.

Statistical Models: Kendall’s tau correlation was tested using the *R cor.test* function. Logistic regressions were run with the R *glm* function *(family=binomial*).

Information about variable creation: HCUP creates these linkages for each state across available ED, inpatient, and ambulatory surgery and services datasets. Several variables of interest were used directly as coded by HCUP (removing N/A values), including age in years (HCUP variable name: AGE), sex (FEMALE), Number of diagnoses (NDX).

Other variables were binarized for clarity of analysis: Race/Ethnicity (RACE; 1=White, 2=Black, 3=Hispanic), Insurance (PAY; 3 = private [vs. all others]), Median household income in zip code (MEDINCSTQ; 1 = lowest quartile [vs. all others]), Urbanicity (PL_UR_CAT4: 1 = Large metropolitan areas with at least 1 million residents [vs. all others]), Disposition (DISPUB04; 1 = routine/home [vs. all others]) Length of Stay (los) was examined as a continuous measure and binarized if admission and discharge were not the same day (los>0 [0=same day]). Similarly Total Charges (TOTCHG) were examined as a continuous and binarized (TOTCHG > $2500).

More information for each variable at:
https://hcup-us.ahrq.gov/db/vars/sedddistnote.jsp?var=**hcupname**

Mental Health and Suicide variables: Clinical characterization was extracted from ICD codes separately for visits/years that use ICD-9 vs. ICD-10. Binary absence/presence variables were created using regular expression search of top diagnoses outlined below. | = or; * % = wildcards

© 2025 Pagliaccio D, et al. *JAMA Network Open*.

<table>
<tr><th>Variable</th><th>ICD-9</th><th>ICD-10</th></tr>
<tr><td>Search Within:</td><td>DX_Visit_Reason1, DX1-5</td><td>I10_DX_Visit_Reason1-3, I10_DX1-5</td></tr>
<tr><td>Mental Health (MH)</td><td>^(29|30|31)</td><td>^(F2|F3|F4|F5|F9|R45|R46)</td></tr>
<tr><td>Internalizing Disorder (depressive, anxiety)</td><td>2962% | 2063% | 300% | 30921 | 3130% | 31323</td><td>F32% | F33% | F34% | F38% | F39% | F40% | F41% | F93%</td></tr>
<tr><td>Externalizing Disorder (ADHD, ODD, CD)</td><td>312% | 314% | 31381</td><td>F90% | F91% | F92%</td></tr>
<tr><td>Search Within:</td><td>ECODE1-7, DX_Visit_Reason1, DX1-15</td><td>I10_DX_Visit_Reason1-3, I10_DX1-15</td></tr>
<tr><td>Suicide Ideation (SI)</td><td>V6284</td><td>R45.851</td></tr>
<tr><td>Suicide Behavior (SB)</td><td>E95$|^E95[0-8]</td><td>T14.91 or self-inflicted injury T*/X*
See, codes on next page ^</td></tr>
<tr><td>Ambiguous potential SB codes</td><td>E98</td><td>T3*4XA|T3*4XD|T3*4XS| T4*4XA|T4*4XD|T4*4XS| T5*4XA|T5*4XD|T5*4XS| T6*4XA|T6*4XD|T6*4XS| T71*4XA|T71*4XD|T71*4XS| T3*4A|T3*4D|T3*4S| T4*4A|T4*4D|T4*4S| T5*4A|T5*4D|T5*4S| T6*4A|T6*4D|T6*4S| T71*4A|T71*4D|T71*4S| Y2*XXA|Y2*XXS|Y2*XXD| Y3*XXA|Y3*XXS|Y3*XXD|T751XXA</td></tr>
<tr><td>Method - Firearm injury (any intent)</td><td>E955, E922</td><td>X72% | X73% | X74% | W33% | W34% | W35% | Y22% | Y23% | Y24%</td></tr>
<tr><td>Method – Ingestion injury</td><td>E950% | E951% | E952%</td><td>T36% | T37% | T38% | T39% | T4% | T5% | T60% | T61% | T62% | T63% | T64% | T65%</td></tr>
<tr><td>Method – Injury with sharp or blunt object</td><td>E956%</td><td>X78% | X79%</td></tr>
<tr><td>Method – Asphyxiation injury (drowning, hanging, strangulation)</td><td>E953% | E954%</td><td>X71% | T71%</td></tr>
<tr><td>EXPLORATORY*</td><td>DX_Visit_Reason1, DX1-5</td><td>I10_DX_Visit_Reason1-3, I10_DX1-5</td></tr>
<tr><td>NSSI</td><td>-</td><td>history of self-harm Z91.5, R45.88 added in 2021</td></tr>
<tr><td>Sleep disorders or disturbance</td><td>327*, 780.5*</td><td>F51*, G47*</td></tr>
<tr><td>Sexual and gender identity disorders</td><td>302.0*, 302.3*, 302.5*, 302.6*, 313.82</td><td>F64*</td></tr>
<tr><td>Housing or family circumstances</td><td>V60*, V61*</td><td>Z59*, Z62*, Z63*</td></tr>
<tr><td>Substance use disorders</td><td>291*, 292*, 303*, 304*, 305*</td><td>F1*</td></tr>
</table>

** ICD codes for substance use disorder, sleep disturbance, sexual or gender identity disorders were each used in <1% and were not examined in main analyses.*

<u>^ SB CODES FROM HCUP (periods removed):</u>

T1491, T360X2A, T361X2A, T362X2A, T363X2A, T364X2A, T365X2A, T366X2A, T367X2A, T368X2A, T3692XA, T370X2A, T371X2A, T372X2A, T373X2A, T374X2A, T375X2A, T378X2A, T3792XA, T380X2A, T381X2A, T382X2A, T383X2A, T384X2A, T385X2A, T386X2A, T387X2A, T38802A, T38812A, T38892A, T38902A, T38992A, T39012A, T39092A, T391X2A, T392X2A, T39312A, T39392A, T394X2A, T398X2A, T3992XA, T400X2A, T401X2A, T402X2A, T403X2A, T404X2A, T405X2A, T40602A, T40692A, T407X2A, T408X2A, T40902A, T40992A, T410X2A, T411X2A, T41202A, T41292A, T413X2A, T4142XA, T415X2A, T420X2A, T421X2A, T422X2A, T423X2A, T424X2A, T425X2A, T426X2A, T4272XA, T428X2A, T43012A, T43022A, T431X2A, T43202A, T43212A, T43222A, T43292A, T433X2A, T434X2A, T43502A, T43592A, T43602A, T43612A, T43622A, T43632A, T43692A, T438X2A, T4392XA, T440X2A, T441X2A, T442X2A, T443X2A, T444X2A, T445X2A, T446X2A, T447X2A, T448X2A, T44902A, T44992A, T450X2A, T451X2A, T452X2A, T453X2A, T454X2A, T45512A, T45522A, T45602A, T45612A, T45622A, T45692A, T457X2A, T458X2A, T4592XA, T460X2A, T461X2A, T462X2A, T463X2A, T464X2A, T465X2A, T466X2A, T467X2A, T468X2A, T46902A, T46992A, T470X2A, T471X2A, T472X2A, T473X2A, T474X2A, T475X2A, T476X2A, T477X2A, T478X2A, T4792XA, T480X2A, T481X2A, T48202A, T48292A, T483X2A, T484X2A, T485X2A, T486X2A, T48902A, T48992A, T490X2A, T491X2A, T492X2A, T493X2A, T494X2A, T495X2A, T496X2A, T497X2A, T498X2A, T4992XA, T500X2A, T501X2A, T502X2A, T503X2A, T504X2A, T505X2A, T506X2A, T507X2A, T508X2A, T50902A, T50992A, T50A12A, T50A22A, T50A92A, T50B12A, T50B92A, T50Z12A, T50Z92A, T510X2A, T511X2A, T512X2A, T513X2A, T518X2A, T5192XA, T520X2A, T521X2A, T522X2A, T523X2A, T524X2A, T528X2A, T5292XA, T530X2A, T531X2A, T532X2A, T533X2A, T534X2A, T535X2A, T536X2A, T537X2A, T5392XA, T540X2A, T541X2A,

© 2025 Pagliaccio D, et al. *JAMA Network Open*.

T542X2A, T543X2A, T5492XA, T550X2A, T551X2A, T560X2A, T561X2A, T562X2A, T563X2A, T564X2A, T565X2A, T566X2A, T567X2A, T56812A, T56892A, T5692XA, T570X2A, T571X2A, T572X2A, T573X2A, T578X2A, T5792XA, T5802XA, T5812XA, T582X2A, T588X2A, T5892XA, T590X2A, T591X2A, T592X2A, T593X2A, T594X2A, T595X2A, T596X2A, T597X2A, T59812A, T59892A, T5992XA, T600X2A, T601X2A, T602X2A, T603X2A, T604X2A, T608X2A, T6092XA, T6102XA, T6112XA, T61772A, T61782A, T618X2A, T6192XA, T620X2A, T621X2A, T622X2A, T628X2A, T6292XA, T63002A, T63012A, T63022A, T63032A, T63042A, T63062A, T63072A, T63082A, T63092A, T63112A, T63122A, T63192A, T632X2A, T63302A, T63312A, T63322A, T63332A, T63392A, T63412A, T63422A, T63432A, T63442A, T63452A, T63462A, T63482A, T63512A, T63592A, T63612A, T63622A, T63632A, T63692A, T63712A, T63792A, T63812A, T63822A, T63832A, T63892A, T6392XA, T6402XA, T6482XA, T650X2A, T651X2A, T65212A, T65222A, T65292A, T653X2A, T654X2A, T655X2A, T656X2A, T65812A, T65822A, T65832A, T65892A, T6592XA, T71112A, T71122A, T71132A, T71152A, T71162A, T71192A, T71222A, T71232A, X710XXA, X711XXA, X712XXA, X713XXA, X718XXA, X719XXA, X72XXXA, X730XXA, X731XXA, X732XXA, X738XXA, X739XXA, X7401XA, X7402XA, X7409XA, X748XXA, X749XXA, X75XXXA, X76XXXA, X770XXA, X771XXA, X772XXA, X773XXA, X778XXA, X779XXA, X780XXA, X781XXA, X782XXA, X788XXA, X789XXA, X79XXXA, X80XXXA, X810XXA, X811XXA, X818XXA

Supplementary Results

Overview. A total of 10,131,432 ED visits were available for 8-12-year-olds across states/years. Of these, 627517 (6%) visits for coded for MH or STB. This included 534654 MH visits (85.2%), 76112 SI visits (12.1%), and 16751 SB visits (2.7%). Common reasons for MH visits included conduct disorder (ICD-9 312, ICD-10 F91), ADHD (314, F90), anxiety (300.00, F41), emotional symptoms (R45)

Age by Sex Differences. In a logistic regression predicting group (SB vs. SI), there was an interaction between age and sex (OR=1.35, z=17.07, p<.001; **Figure S2**), such that sex differences in suicide risk became more pronounced from younger to older ages. Specifically, SI and SB visits were more common among females and this disparity increased over ages; at age 8: 28% female for SI and 35% for SB visits (OR=1.39) vs. at age 12: 63% female for SI and 82% of SB visits (OR=2.80).

Neighborhood Social Determinants of Health. N= 506,079 visits could be linked via zip code to SDI scores (n=11564 unique zip codes). Zip codes were unavailable for Arkansas and Massachusetts in all years and Maryland in 2013-2017 and thus these visits were excluded from zip code analyses. SDI, ADI, and COI scores were highly correlated in this population (r>|.89|). As with SDI scores (Table 1), STB cases were associated with less deprivation/more opportunity than MH cases on the ADI and COI.
ADI: MH = 55.36 (29.4) vs. STB = 50.95 (28.57), t=-39.40, p<.001, Cohen's d= -0.15
COI: MH = 43.22 (28.47) vs. STB = 48.85 (27.60), t=52.23, p<.001, Cohen's d=0.20

© 2025 Pagliaccio D, et al. *JAMA Network Open*.

eFigure 1. Workflow

1. Stack (union) all state/year datasets by ICD version (ICD-9 2010-2015 Q1-Q3 vs. ICD-10 2015 Q4-2020)
2. Filter where AGE >= 8 and AGE <= 12
3. Filter where DX1 != NULL
4. Label ICD code
5. Group ifelse SB, SI, MH, Other
6. Stack (union) ICD-9 and ICD-10 data together
7. Aggregate (summarize) by state and year to count total number of visits
8. Filter where GROUP == MH | SI | SB (remove other diagnoses)
9. Aggregate (summarize) by ID to count number of visits
10. Aggregate (summarize) by ID to count number of SI/SB visits
11. Join (merge) main data with aggregated summaries and zip code data (SDI, ADI, COI)
12. Clean final variables, binarize, remove NULL
13. Sort by ID and visit order/timing

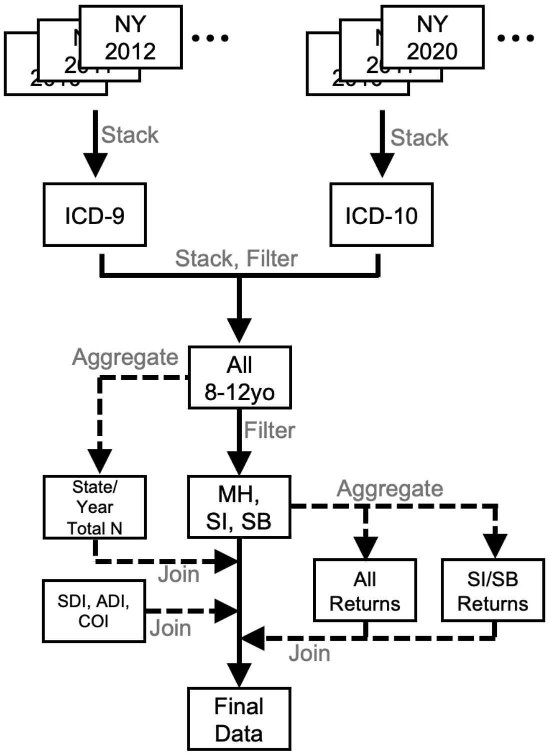

© 2025 Pagliaccio D, et al. *JAMA Network Open*.

eFigure 2. Count of Suicide-Related Visits Per State per Year

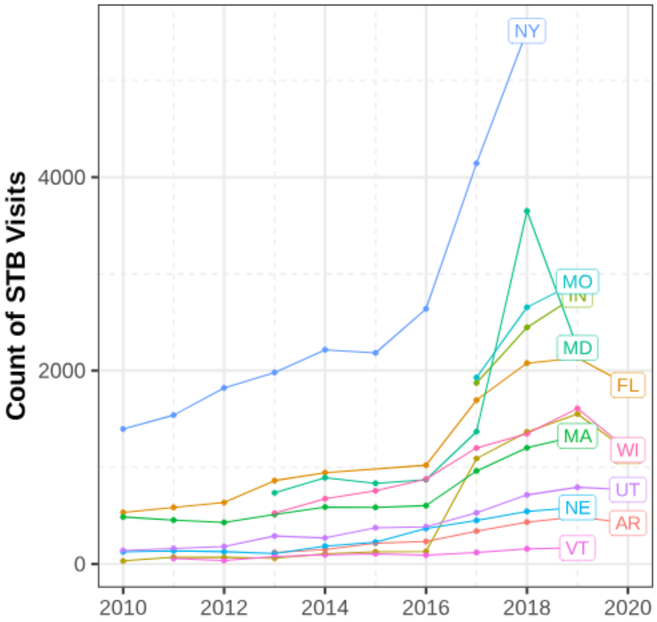

Note: ED visits for suicide thoughts and behaviors (STB) were tabulated by state and year. See eTable 1 for additional breakdown.

© 2025 Pagliaccio D, et al. *JAMA Network Open*.

eFigure 3. Rates of Code Groups by State

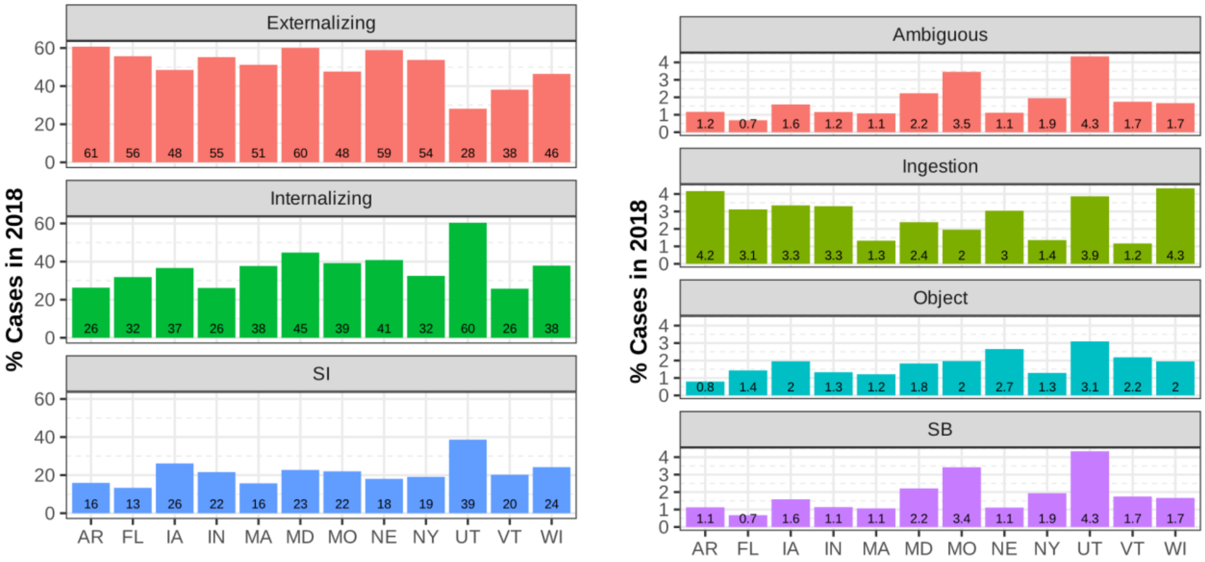

Note. Key ICD-10 code groups are displayed by state. All data indicate the percentage of total visits per state in 2018 endorsing a given code. 2018 was examined an examplar year as all states contirbuted data to HCUP in this year and all codes were ICD-10 version. Cases may include multiple code groups (i.e., these are not mutually exclusive and thus percentages can add to >100% per year). See eMethods above for coding details.

-The left panel shows the rates of cases with externalizing diagnoses, internalizing diagnoses, and suicide ideation (SI) codes.

-The right panel shows the rates of cases with ambigous suicide behavior codes, ingestion injury codes, object injury codes, and suicide behavior (SB) codes.

© 2025 Pagliaccio D, et al. *JAMA Network Open*.

eFigure 4: Age x Sex Differences

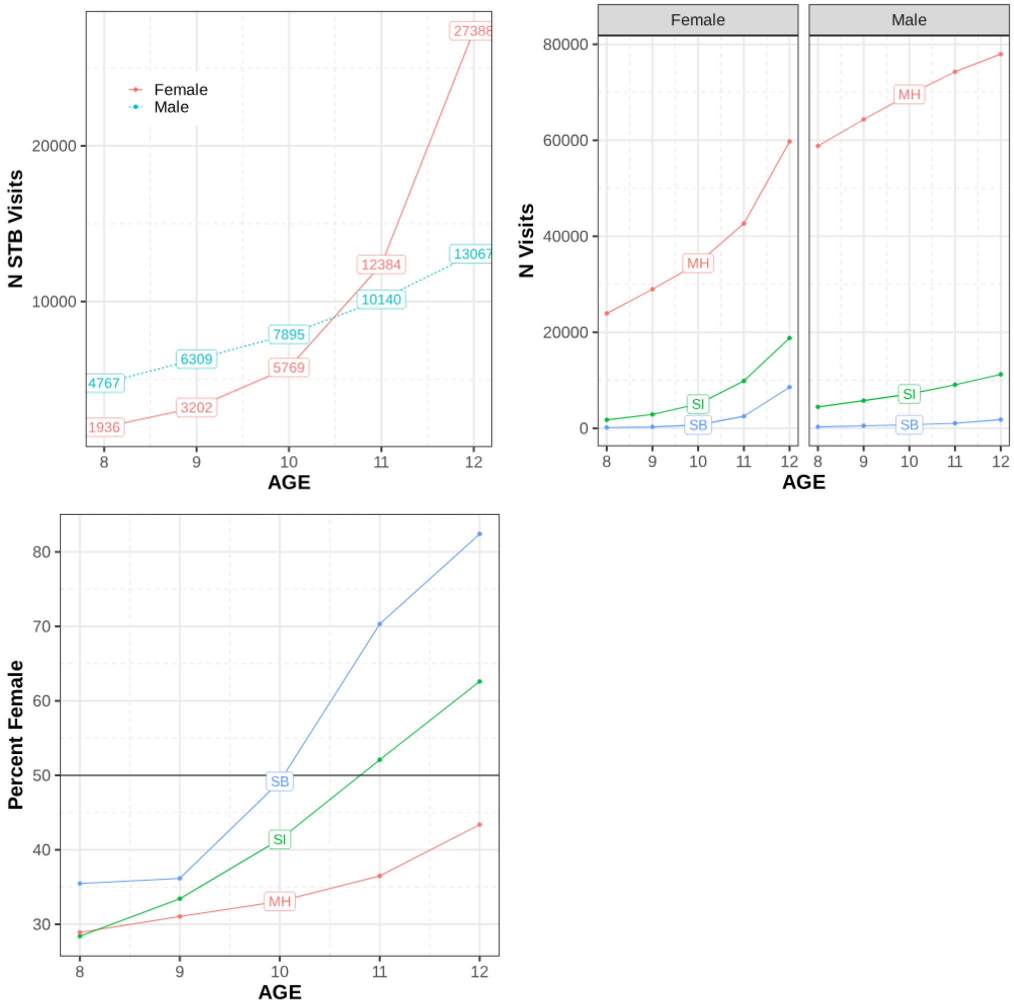

Note: ED visits were tabulated by age (years) and grouping: non-suicide mental health (MH) cases relative to suicide thoughts and behaviors (STB) or split by suicide ideation (SI) and suicide behavior (SB).

- The top left panel displays the count of STB visits split by age and sex.
- The top right panel displays the count of visits by age and three groups split by sex.
- The bottom panel displays the percent of visits for female vs. boys per age and group.

© 2025 Pagliaccio D, et al. *JAMA Network Open*.

eFigure 5. Demographic Trends in Suicide-Related Visits over Time

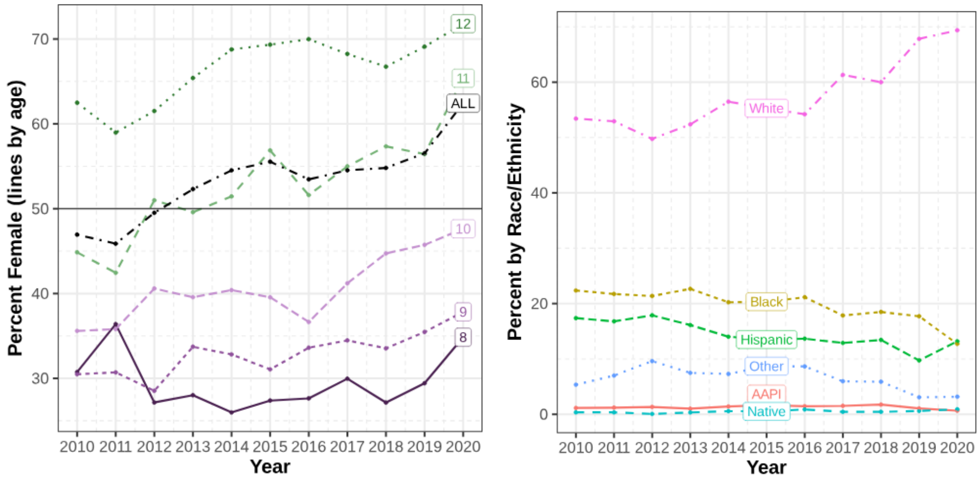

Note: ED visits for suicide thoughts and behaviors (STB) were tabulated and split by demographics by year (percentages add to 100% in each year).

- The left panel displays the percent of STB visits for female patients per age group and per year; the black dashed/dotted line indicates the overall percentage across ages.
- The right panel shows the percent of STB visits by EHR coding of race/ethnicity per year. These were coded as mutually exclusive options in HCUP data.

© 2025 Pagliaccio D, et al. *JAMA Network Open*.

eFigure 6. Visit Timing

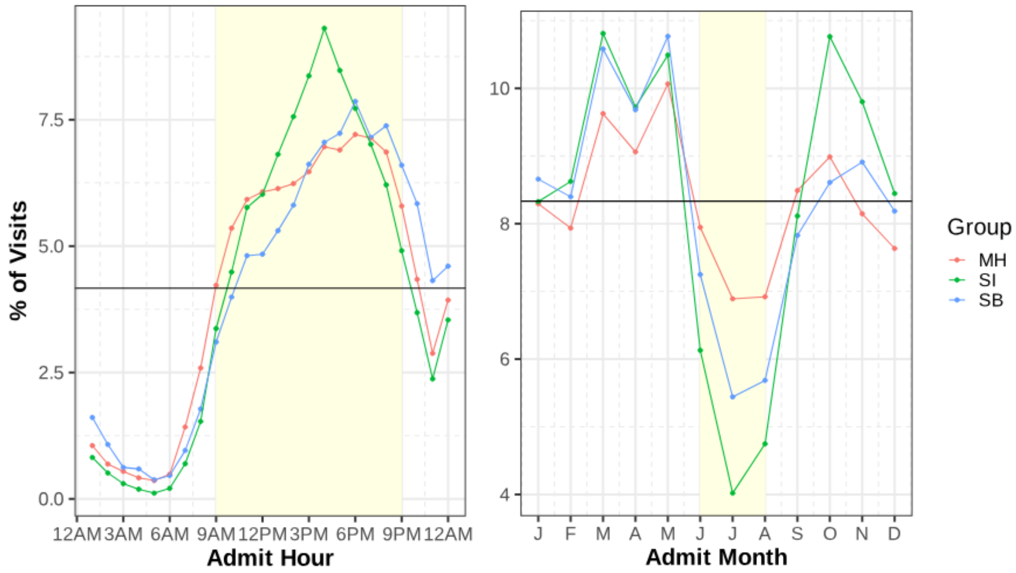

Note: The percent visits within group (mental health [MH], suicide ideation [SI], suicide behavior [SB]) are presented split by time bin. For example, the percentages along the MH line (red) will sum to 100% of all MH visits.

- The left panel indicates the percent of visits by hour of admission time (rounded to then earest hour). The horizontal line indicates an even distribution across time (1/24); the yellow shaded area indicated 9am-9pm as presented in Tables 1, S1.
- The right panel indicates the percent of visits by month of admission date. The horizontal line indicates an even distribution across time (1/12); the yellow shaded area indicated summer months (not school months) as presented in Tables 1, S1.

© 2025 Pagliaccio D, et al. *JAMA Network Open*.

eFigure 7. Timing of ED Return Visits

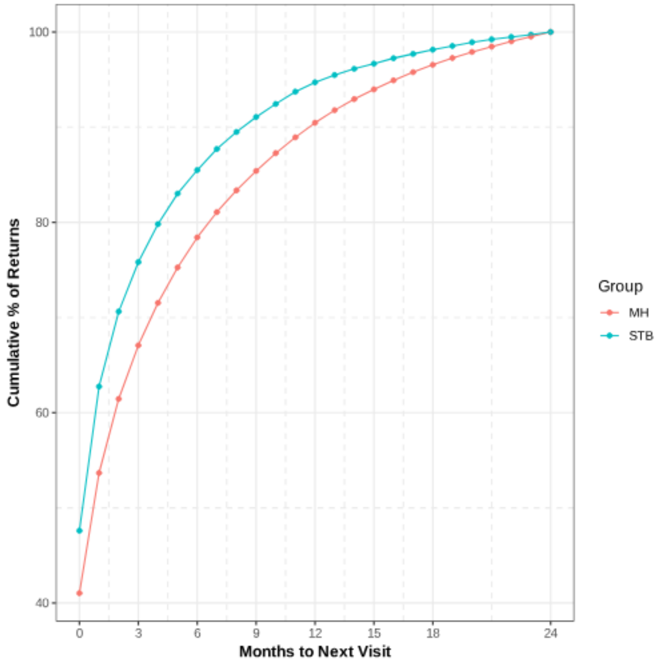

Note. Visits with a subsequent return visit were grouped based on mental health (MH) or suicide thoughts or behaviors (STB) at the initial visit. The number of months until the subsequent visit were examined and plotted as a cumulative proportion within the following 24 years. For example, of cases with a subsequent return, 40-50% are within one month of discharge.

© 2025 Pagliaccio D, et al. *JAMA Network Open*.

eTable 1. Count of Visits Per State per Year

State	2010	2011	2012	2013	2014	2015	2016	2017	2018	2019	2020
AR	NA	NA	NA	3163 / 49445 (6%)	3086 / 50063 (6%)	2917 / 50484 (6%)	2320 / 51506 (5%)	2393 / 51831 (5%)	2399 / 48884 (5%)	2839 / 50061 (6%)	2046 / 35700 (6%)
FL	9261 / 280062 (3%)	10057 / 290720 (3%)	11057 / 324212 (3%)	10991 / 324803 (3%)	12660 / 352732 (4%)	NA	11016 / 392374 (3%)	12199 / 392679 (3%)	13420 / 396905 (3%)	15296 / 401167 (4%)	10933 / 236710 (5%)
IA	1312 / 41374 (3%)	1276 / 44080 (3%)	1470 / 43545 (3%)	1230 / 41535 (3%)	1353 / 42633 (3%)	1720 / 43793 (4%)	1640 / 42923 (4%)	4216 / 45494 (9%)	4660 / 42594 (11%)	4933 / 41759 (12%)	3449 / 28786 (12%)
IN	NA	NA	NA	NA	NA	NA	NA	9146 / 111661 (8%)	10085 / 103191 (10%)	10629 / 104396 (10%)	NA
MA	5564 / 94795 (6%)	5668 / 95542 (6%)	5766 / 95882 (6%)	6195 / 91092 (7%)	6290 / 91131 (7%)	6875 / 92778 (7%)	6705 / 92429 (7%)	6847 / 92686 (7%)	6939 / 88979 (8%)	7165 / 85974 (8%)	NA
MD	NA	NA	NA	6351 / 84285 (8%)	6603 / 84376 (8%)	6720 / 82900 (8%)	5799 / 81663 (7%)	6484 / 79948 (8%)	14700 / 112962 (13%)	9435 / 72036 (13%)	NA
MO	NA	NA	NA	NA	NA	NA	NA	9850 / 149114 (7%)	10790 / 135410 (8%)	10671 / 130298 (8%)	NA
NE	1200 / 20850 (6%)	1360 / 21442 (6%)	1465 / 22088 (7%)	1684 / 20367 (8%)	1992 / 21924 (9%)	2198 / 22761 (10%)	2226 / 23689 (9%)	2259 / 23390 (10%)	2600 / 23396 (11%)	2560 / 23068 (11%)	NA
NY	17844 / 279273 (6%)	20052 / 292229 (7%)	21148 / 289158 (7%)	21663 / 277662 (8%)	22912 / 275860 (8%)	21416 / 276275 (8%)	19625 / 282293 (7%)	23143 / 278424 (8%)	26473 / 271935 (10%)	NA	NA
UT	1442 / 26434 (5%)	1162 / 27397 (4%)	1164 / 27643 (4%)	1243 / 27214 (5%)	1443 / 28802 (5%)	1576 / 29502 (5%)	1349 / 30864 (4%)	1519 / 29465 (5%)	1681 / 28724 (6%)	2217 / 28731 (8%)	1939 / 21505 (9%)
VT	NA	544 / 9181 (6%)	435 / 9432 (5%)	490 / 8880 (6%)	483 / 8371 (6%)	516 / 8533 (6%)	452 / 8521 (5%)	566 / 8493 (7%)	687 / 8175 (8%)	642 / 7930 (8%)	NA
WI	NA	NA	NA	5600 / 61076 (9%)	6743 / 65288 (10%)	6525 / 66778 (10%)	5856 / 68998 (8%)	6215 / 70065 (9%)	4923 / 66653 (7%)	5620 / 65773 (9%)	4076 / 44538 (9%)

Note. Each cell indicates the number of mental health and suicide-related visits / total visits for any cause for 8-12-year-olds (% of MH+STB / total) per state and per year.
NA = not available

© 2025 Pagliaccio D, et al. *JAMA Network Open*.

eTable 2. Characteristics of Suicide-Related Visits

Variable	SI Visits (N=76112)	SB Visits (N=16751)	SI>SB Statistic	Effect Size
Sociodemographics				
Age (years)	10.76 (1.3)	11.35 (1.02)	t=64.30	d=0.50
Sex (female)	38399 (50.45%)	12280 (73.31%)	χ2=2891.76	OR=2.70
Race (White)	42574 (58.43%)	9815 (61.95%)	χ2=66.57	OR=1.16
Race (Black)	14014 (19.23%)	2440 (15.4%)	χ2=126.25	OR=0.76
Ethnicity (Hispanic)	9371 (12.86%)	2190 (13.82%)	χ2=10.54	OR=1.09
Insurance (private) *	24913 (32.77%)	5630 (33.64%)	χ2=4.66	OR=1.04
Median household income in zip code (lowest quartile)	24170 (32.02%)	5084 (30.58%)	χ2=12.92	OR=0.94
SDI (1-100)	53.52 (28.85)	51.2 (28.26)	t=-8.85	d=-0.08
Urbanicity (1M+ residents)	38489 (50.65%)	7680 (45.9%)	χ2=123.65	OR=0.83
Clinical Factors				
Number of diagnoses	2.76 (1.68)	3.79 (2.29)	t=54.97	d=0.51
Internalizing Diagnosis	31527 (41.42%)	6373 (38.05%)	χ2=64.65	OR=0.87
Externalizing Diagnosis	17950 (23.58%)	1933 (11.54%)	χ2=1182.86	OR=0.42
Length of ED Stay (days) *	0.4 (0.74)	0.41 (0.69)	t=2.38	d=0.02
Length of ED Stay (>1 day)	24593 (32.38%)	5919 (35.37%)	χ2=55.52	OR=1.14
Disposition (discharge home)	43108 (61.79%)	8489 (54.64%)	χ2=271.73	OR=0.74
Total Charges ($)	2356.02 (2435.09)	3234.77 (3373.85)	t=30.66	d=0.30
Total Charge (>$2500)	21406 (30.68%)	7255 (46.95%)	χ2=1499.75	OR=2.00
High Complexity Visit (*ns*)	28116 (36.94%)	6155 (36.74%)	χ2=0.22	OR=0.99
Temporality of Admission				
On Weekday (M–F)	65881 (86.57%)	13407 (80.04%)	χ2=468.53	OR=0.62
Daytime hours 9am–9pm	36564 (78.41%)	6956 (68.72%)	χ2=436.02	OR=0.60
School Months (Sept–May)	49021 (85.1%)	10021 (81.62%)	χ2=93.29	OR=0.78
School Day (weekday Sept–May)	42812 (71.31%)	8147 (61.73%)	χ2=468.75	OR=0.65

Note. Sociodemographic, clinical, and temporal characteristics of pediatric visits are presented for the subset of visits with suicide ideation (SI) vs. suicide behavior (SB).
Continuous variables are summarized with their group mean and standard deviation. Group differences are tested via t-test with their accompanying Cohen's d effect size. Categorical variables are summarized by count and subgroup percentage. Group differences are tested via chi-squared test (χ2) and accompanying odds ratio (OR). Positive Cohen's d and OR>1 indicate higher score/more prevalence in the SB > SI group.
All differences between groups were p<.001 significant, except those noted in gray text and marked as *p<.05 or non-significant (ns).
Missing data per variable: sex (n=6), race/ethnicity (n=4152), insurance (n=114), income (n=749), social deprivation index (SDI; n=14926), urbanicity (n=138), length of stay (n=166), disposition (n=7566), total charges (n=7647), admission day (n=7), admission time (n=36109), admission month (n=22985)

© 2025 Pagliaccio D, et al. *JAMA Network Open*.

eTable 3. Characteristics of SI Visits with Ambiguous Behavior Codes

Variable	SI only (N= 73847)	SI + Ambiguous Code (N=2265)	SI > SI+ambig Statistic	Effect Size
Sociodemographics				
Age (years)	10.75 (1.3)	11.2 (1.09)	t=19.39	d=0.38
Sex (female)	36767 (49.79%)	1632 (72.18%)	χ2=439.10	OR=2.62
Race (White) **	41242 (58.33%)	1332 (61.61%)	χ2=9.16	OR=1.15
Race (Black) **	13655 (19.31%)	359 (16.6%)	χ2=9.73	OR=0.83
Ethnicity (Hispanic) *ns*	9082 (12.84%)	289 (13.37%)	χ2=0.47	OR=1.05
Insurance (private) *ns*	24167 (32.77%)	746 (32.95%)	χ2=0.03	OR=1.01
Median household income in zip code (lowest quartile) **	23518 (32.11%)	652 (28.99%)	χ2=9.62	OR=0.86
SDI (1-100)	53.66 (28.87)	49.49 (27.99)	t=-6.61	d=-0.15
Urbanicity (1M+ residents)	37259 (50.54%)	1230 (54.33%)	χ2=12.49	OR=1.16
Clinical Factors				
Number of diagnoses	2.7 (1.64)	4.49 (2.19)	t=38.52	d=0.92
Internalizing Diagnosis	30169 (40.85%)	1358 (59.96%)	χ2=329.71	OR=2.17
Externalizing Diagnosis *ns*	17435 (23.61%)	515 (22.74%)	χ2=0.88	OR=0.95
Length of ED Stay (days)	0.39 (0.73)	0.53 (0.97)	t=6.71	d=0.16
Length of ED Stay (>1 day)	23696 (32.15%)	897 (39.62%)	χ2=55.60	OR=1.38
Disposition (discharge home)	42025 (62.15%)	1083 (50.51%)	χ2=118.74	OR=0.62
Total Charges ($)	2346.54 (2435)	2715.34 (2411.91)	t=6.39	d=0.15
Total Charge (>$2500)	20621 (30.34%)	785 (43.78%)	χ2=147.82	OR=1.79
High Complexity Visit	27122 (36.73%)	994 (43.89%)	χ2=48.03	OR=1.35
Temporality of Admission				
On Weekday (M–F) *ns*	63950 (86.61%)	1931 (85.25%)	χ2=3.34	OR=0.89
Daytime hours 9am–9pm	35727 (78.51%)	837 (74.2%)	χ2=11.84	OR=0.79
School Months (Sept–May) *ns*	47534 (85.12%)	1487 (84.73%)	χ2=0.17	OR=0.97
School Day (weekday Sept–May) *	41529 (71.36%)	1283 (69.77%)	χ2=2.14	OR=0.93

Note. Sociodemographic, clinical, and temporal characteristics of pediatric visits are presented for the subset of visits with suicide ideation (SI) split by the absence/present of ambiguous ICD codes that may indicate potential suicide behaviors (e.g. injury with undetermined intent). Continuous variables are summarized with their group mean and standard deviation. Group differences are tested via t-test with their accompanying Cohen's d effect size. Categorical variables are summarized by count and subgroup percentage. Group differences are tested via chi-squared test (χ2) and accompanying odds ratio (OR). Positive Cohen's d and OR>1 indicate higher score/more prevalence in the SB > SI group.
All differences between groups were p<.001 significant, except those noted in gray text and marked as *p<.05, **p<.01, or non-significant (ns).
Missing data per variable: sex (n=6), race/ethnicity (n=3245), insurance (n=73), income (n=623), social deprivation index (SDI; n=12540), urbanicity (n=120), length of stay (n=150), disposition (n=6351), total charges (n=6347), admission day (n=7), admission time (n=29480), admission month (n=16079).

© 2025 Pagliaccio D, et al. *JAMA Network Open*.

eTable 4. Characteristics of Suicide Behavior Visits by Method

Variable	Ingestion (N= 6939)	Object (N=6334)	Ingestion > Object Statistic	Effect Size
Sociodemographics				
Age (years)	11.55 (0.85)	11.44 (0.89)	t=-7.47	d=-0.13
Sex (female)	5781 (83.31%)	4991 (78.8%)	χ2=43.84	OR=0.74
Race (White) *ns*	4089 (63.02%)	3804 (62.86%)	χ2=0.03	OR=0.99
Race (Black)	1036 (15.97%)	715 (11.81%)	χ2=44.62	OR=0.71
Ethnicity (Hispanic)	842 (12.98%)	940 (15.53%)	χ2=16.55	OR=1.23
Insurance (private)	2591 (37.37%)	2053 (32.46%)	χ2=34.87	OR=0.81
Median household income in zip code (lowest quartile) (ns)	1961 (28.49%)	1957 (31.08%)	χ2=10.49	OR=1.13
SDI (1-100)	48.55 (27.57)	51.9 (28.52)	t=6.36	d=0.12
Urbanicity (1M+ residents)	2970 (42.84%)	3105 (49.08%)	χ2=51.48	OR=1.29
Clinical Factors				
Number of diagnoses	3.47 (2.14)	4.34 (2.29)	t=22.62	d=0.39
Internalizing Diagnosis	2497 (35.99%)	2814 (44.43%)	χ2=97.96	OR=1.42
Externalizing Diagnosis	587 (8.46%)	747 (11.79%)	χ2=40.35	OR=1.45
Length of ED Stay (days)	0.5 (0.69)	0.35 (0.68)	t=-12.57	d=-0.22
Length of ED Stay (>1 day)	3009 (43.39%)	1873 (29.58%)	χ2=270.56	OR=0.55
Disposition (discharge home)	2746 (41.93%)	3975 (68.86%)	χ2=896.07	OR=3.06
Total Charges ($)	4311.21 (3518.84)	2247.7 (2327.54)	t=-38.63	d=-0.69
Total Charge (>$2500)	4469 (68.75%)	1666 (29.17%)	χ2=1904.29	OR=0.19
High Complexity Visit	3135 (45.18%)	1876 (29.62%)	χ2=340.56	OR=0.51
Temporality of Admission				
On Weekday (M–F)	5392 (77.71%)	5237 (82.68%)	χ2=51.07	OR=1.37
Night hours 9pm–9am	2308 (59.27%)	2953 (74.53%)	χ2=206.11	OR=2.01
School Months (Sept–May)	4023 (80.09%)	3872 (83.7%)	χ2=20.85	OR=1.28
School Day (weekday Sept–May)	3150 (57.8%)	3290 (66.53%)	χ2=83.53	OR=1.45

Note. Sociodemographic, clinical, and temporal characteristics of pediatric visits are presented for the subset of visits with suicide behaviors (SB) split by injury method. Only cases with one injury code are examined (excluded those with multiple codes, no injury codes, or other rarer types; for clarity).

Continuous variables are summarized with their group mean and standard deviation. Group differences are tested via t-test with their accompanying Cohen's d effect size. Categorical variables are summarized by count and subgroup percentage. Group differences are tested via chi-squared test (χ2) and accompanying odds ratio (OR). Positive Cohen's d and OR>1 indicate higher score/more prevalence in the object > ingestion group.

All differences between groups were p<.001 significant, except those noted in gray text (non-significant [ns]).

Missing data per variable: race/ethnicity (n=733), insurance (n=15), income (n=93), social deprivation index (SDI; n=1848), urbanicity (n=14), length of stay (n=7), disposition (n=951), total charges (n=1061), admission time (n=5417), admission month (n=3624).

© 2025 Pagliaccio D, et al. *JAMA Network Open*.

eTable 5. Sensitivity Analyses for State Differences

Variable	Table 2: Model 1 aOR [99% CI]	*GLMER aOR*	*LOO aOR range*
STB vs. MH	9.71 [9.66–9.76]	8.86	8.95–11.16
Age (years)	1.06 [1.04–1.08]	1.06	1.05–1.07
Sex (female)	1.33 [1.29–1.37]	1.33	1.29–1.33
Race (White)	1.05 [0.98–1.12]	1.10	0.99–1.13
Race (Black)	1.03 [0.95–1.11]	1.06	0.95–1.10
Ethnicity (Hispanic)	0.72 [0.63–0.81]	0.85	0.64–0.85
Insurance (private)	0.79 [0.74–0.84]	0.74	0.77–0.81
Urbanicity	0.68 [0.64–0.72]	0.65	0.58–0.73
Internalizing Diagnosis	1.37 [1.33–1.41]	1.34	1.29–1.41
Externalizing Diagnosis	1.39 [1.35–1.43]	1.29	1.32–1.43
Length of ED Stay	1.20 [1.15–1.25]	1.21	1.13–1.27
Disposition (home)	0.71 [0.66–0.76]	0.63	0.68–0.76
High Complexity Visit	1.13 [1.08–1.18]	1.13	1.00–1.22

Note: Main text logistic regression models tested whether each visit was followed by a subsequent visit for suicide thoughts or behaviors (STB) within the next 1 year. Cases were removed with listwise deletion for missing covariates. The main model examined n=537,043 visits, with 3.73% having a return visit for STB within 1 year.

- The main text results are shown for comparison (Table 2: Model 1 column), adjusted odds ratios (aOR) and 99% confidence intervals (CI) are shown.
- The GLMER column displays aOR results from a generalized logistic mixed-effects model with a random intercept for state.
- The LOO columns displays leave-one-state-out cross-validation (12 iterations of logistic regions) results; the min–max range of aOR are displayed.

© 2025 Pagliaccio D, et al. *JAMA Network Open*.
