# Supplementary material for: Childhood Suicide Risk in the Emergency Department
Source: JAMA Netw Open. 2025 Jul 22;8(7):e2522591. doi: 10.1001/jamanetworkopen.2025.22591 (PMC12284741; doi:10.1001/jamanetworkopen.2025.22591)
Supplement: Supplement 2. — Data Sharing Statement [file jamanetwopen-e2522591-s002.pdf]

Data Sharing Statement

Data
Data available: No

Additional Information
Explanation for why data not available: Data are made available by HCUP by DUA only
